# A Systems Biology Approach for Studying Heterotopic Ossification: Proteomic Analysis of Clinical Serum and Tissue Samples

**DOI:** 10.1016/j.gpb.2018.04.006

**Published:** 2018-07-24

**Authors:** Erin L. Crowgey, Jennifer T. Wyffels, Patrick M. Osborn, Thomas T. Wood, Laura E. Edsberg

**Affiliations:** 1 Nemours Biomedical Research, Nemours Alfred I. duPont Hospital for Children, Wilmington, DE 19803, USA; 2 Natural and Health Sciences Research Center, Center for Wound Healing Research, Daemen College, Amherst, NY 14226, USA; 3 Department of Computer and Information Sciences, Center for Bioinformatics and Computational Biology, University of Delaware, Newark, DE 19711, USA; 4 San Antonio Military Medical Center, San Antonio, TX 78219, USA

**Keywords:** Heterotopic ossification, Proteomics, Runt-related transcription factor 2, Extracellular matrix organization, Keratinization

## Abstract

**Heterotopic ossification** (HO) refers to the abnormal formation of bone in soft tissue. Although some of the underlying processes of HO have been described, there are currently no clinical tests using validated biomarkers for predicting HO formation. As such, the diagnosis is made radiographically after HO has formed. To identify potential and novel biomarkers for HO, we used isobaric tags for relative and absolute quantitation (iTRAQ) and high-throughput antibody arrays to produce a semi-quantitative **proteomics** survey of serum and tissue from subjects with (HO^+^) and without (HO^−^) heterotopic ossification. The resulting data were then analyzed using a systems biology approach. We found that serum samples from subjects experiencing traumatic injuries with resulting HO have a different proteomic expression profile compared to those from the matched controls. Subsequent quantitative ELISA identified five blood serum proteins that were differentially regulated between the HO^+^ and HO^−^ groups. Compared to HO^−^ samples, the amount of insulin-like growth factor I (IGF1) was up-regulated in HO^+^ samples, whereas a lower amount of osteopontin (OPN), myeloperoxidase (MPO), **runt-related transcription factor 2** (RUNX2), and growth differentiation factor 2 or bone morphogenetic protein 9 (BMP-9) was found in HO^+^ samples (Welch two sample *t*-test; *P* < 0.05). These proteins, in combination with potential serum biomarkers previously reported, are key candidates for a serum diagnostic panel that may enable early detection of HO prior to radiographic and clinical manifestations.

## Introduction

Heterotopic ossification (HO), the abnormal formation of mature lamellar bone in nonosseous (soft) tissue, is a significant problem for wounded soldiers that have survived high energy blast injuries [1], [2]. A recent study on soldiers from Operation Enduring Freedom and Operation Iraqi Freedom reveals that the highest risk of HO follows amputation from a blast mechanical injury, with HO accounting for >60% combat-related extremity injuries [1], [3]. Of interest, in the military population, formation of HO is associated with chronic pain, prostheses not fitting properly, joint ankylosis, functionality limitations, longer rehabilitation, and substantial morbidity [3]. Additionally, HO occurs post-trauma in elective hip arthroplasty, externally fixed distal humerus fractures (42%), spinal cord injury (SCI), and closed brain injury in civilian populations [4].

Treatment regimens for HO are limited by a lack of understanding of the cellular events that contribute to disease onset. Although non-steroidal anti-inflammatory (NSAID) drugs and radiation therapy used prophylactically can be effective as a treatment for HO, many patients need at least one surgical excision of ectopic bone [5]. Multiple diagnoses, including hemostasis and polytrauma, often present in combat casualties, make these prophylactic treatments contraindicated, and currently there are no pharmaceutical treatments yet approved by the United States Federal Drug Administration to treat HO once present [5].

Recent technological advancements in the field of mass spectrometry (MS) have enhanced the ability to perform proteomic analysis of biological samples and facilitate the identification of disease biomarkers [6]. High-throughput MS techniques, such as isobaric tags for relative and absolute quantitation (iTRAQ), enable a global analysis of the proteome differences between biological samples. This approach enables a wholistic data driven experimental design that does not require *a priori* specification of protein targets. The objective of this study was to collect and integrate serum and tissue proteomes from HO^+^ and HO^−^ subjects, in order to identify proteins and pathways that are dysregulated in the disease state and provide insight into potential biomarkers for early disease detection and monitoring.

## Results

### Subject demographics and experimental workflow

**Figure 1:**
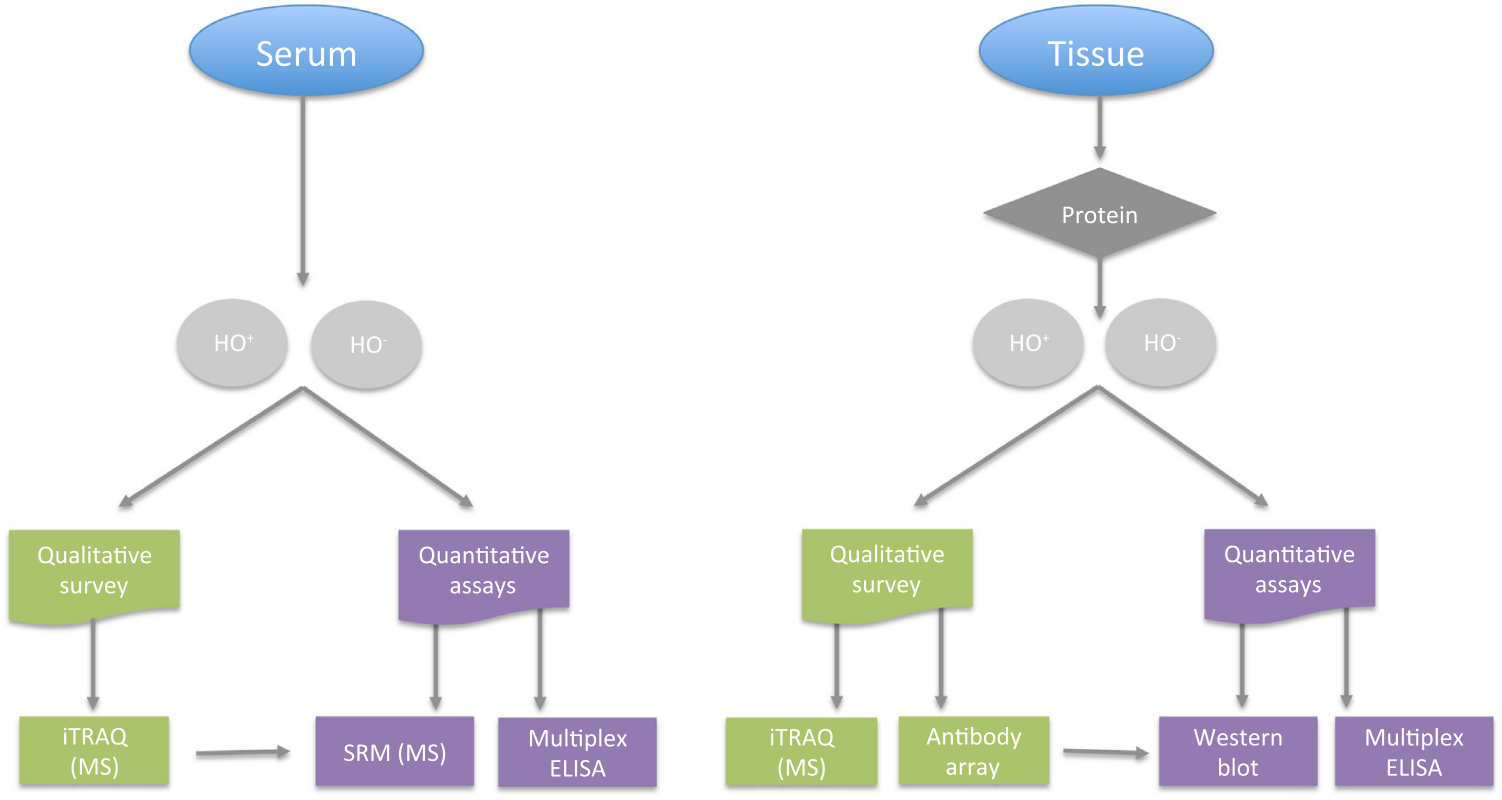
**Overview of the workflow for HO sample analyses** A hierarchical proteomic analysis was applied to the serum (**A**) or tissue (**B**) samples from HO^+^ and HO^−^ subjects, by combining qualitative or semi-quantitative antibody arrays and MS shotgun proteomic surveys to detect novel potential biomarkers. Antibody-independent SRM MS analysis and antibody-dependent ELISA or Western blot quantitative assays were subsequently performed to validate each potential biomarker. HO, heterotopic ossification; MS, mass spectrometry; SRM, selected reaction monitoring; iTRAQ, isobaric tags for relative and absolute quantitation.

Forty-four subjects were enrolled in this study. Tissue samples were collected from 42 subjects with 41 tissue samples having matched serum samples. HO^−^ subjects (*n* = 33) aged 22–83 years, whereas HO^+^ subjects (*n* = 10) aged 22–40 years. The HO^−^ tissue samples were acquired mainly through total hip arthroplasty, whereas the HO^+^ samples were acquired via hip revision or HO excision (Table 1). Serum and tissue samples were analyzed following the workflow shown in Figure 1.

**Table 1:** **Subject demographics**

	**HO**^−^			**HO^+^**		
	**M**	**F**	**Age range (mean)**	**M**	**F**	**Age range (mean)**
Subjects						
With serum samples	18	13	22–83 (54)	9	1	22–40 (29)
With tissue samples	20	13	22–83 (52)	8	1	22–40 (28)

Injury etiology						
Total hip arthroplasty	11	8	28–83 (59)			
Open reduction and internal fixation	6	3	25–64 (45)			
Hip revision		3	45–62 (56)	1	1	36–40 (38)
HO excision				8		22–31 (26)
Others	3		22–36 (29)			

*Note*: HO, heterotopic ossification; M, male; F, female.

### High-throughput screening and Western blot validation

To identify potential markers for HO, high-throughput antibody microarrays were used for an initial screening of 877 cell signaling proteins by comparing the HO^−^ and HO^+^ groups. >200 protein candidates had a 50% or greater difference in spot intensity between the pooled HO^−^ and pooled HO^+^ serum samples. These 200 candidates were further filtered to remove proteins with high variations for duplicate measurements, flagged protein spots with irregular margins, or proteins with a global normalized score <800. As a result, 67 targets were retained for further validation and the top 18 proteins based on Z-ratios were subjected to Western blotting analysis.

Western blots validated the microarray data for phosphorylated GRB2-associated-binding protein 1 (Gab1 Y627) and apoptosis regulator BAX between the pooled HO^+^ and pooled HO^−^ samples. However, spot intensity was weak for both Gab1 Y627 and BAX, and the BAX antibody had strong non-specific cross reactivity. Weak binding and cross-reactivity in addition to large sample volume requirements diminished the utility of Western blotting and as a result no additional serum samples were analyzed using this technique.

**Figure 2:**
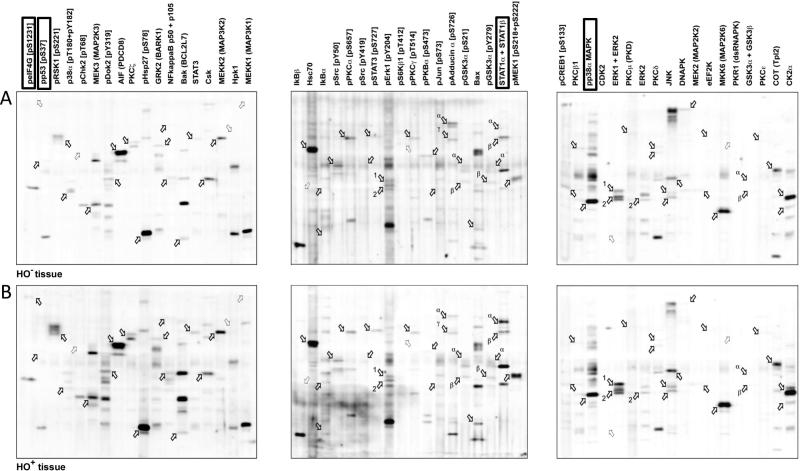
**Western blot analysis to identify biomarkers from tissue samples** Pooled HO^−^ (**A**) or HO^+^ (**B**) tissue samples were tested for differential expression of 54 targets selected from semi-quantitative antibody arrays. Dark outlined arrows indicate the expected positions of the target proteins detected by their respective antibodies, whereas light outlined arrows indicate the migration positions of target proteins that were not visualized.

Using antibody arrays and the filtering criteria described for serum (above), 54 proteins were selected for Western blot validation using extracted proteins from pooled HO^+^ and pooled HO^−^ tissue samples. The levels of phosphorylated (pS1231) eukaryotic translation initiation factor 4 (eIF4G) and signal transducer and activator of transcription 1a (STAT1a) were increased in HO^+^ tissue samples compared to HO^−^ samples, whereas levels of p38a MAPK and phosphorylated (pS37) p53 were down-regulated in HO^+^ compared to the HO^−^ samples (Figure 2). All other proteins showed comparable expression between HO^+^ and HO^−^ samples. Proteins with demonstrated differences in abundance between pooled HO^+^ and pooled HO^−^ tissue samples were further tested on individual, non-pooled samples; however, the results were heterogeneous within the HO^−^ and HO^+^ cohorts, highlighting the complexity and limitations of scaling results from pooled analyses to the individual level.

### Global proteomic survey identified HO-related proteins

iTRAQ was used to generate a semi-quantitative global proteomics survey of serum and tissue from subjects with and without HO. In total, 1220 and 3770 unique proteins were assigned UniProtKB Accessions across the biological and technical replicates of iTRAQ experiments for serum samples and tissue samples, respectively. Protein abundance obtained from iTRAQ experiments was expressed as a ratio of protein levels in HO^−^ samples against that in HO^+^ samples for each protein quantified. There were 648 proteins that were quantified in both serum and tissue samples. The ratios for the majority of proteins identified in serum (85%) and tissue (92%) were close to 1.0, indicating similar abundances between HO^−^ and HO^+^ samples.

**Figure 3:**
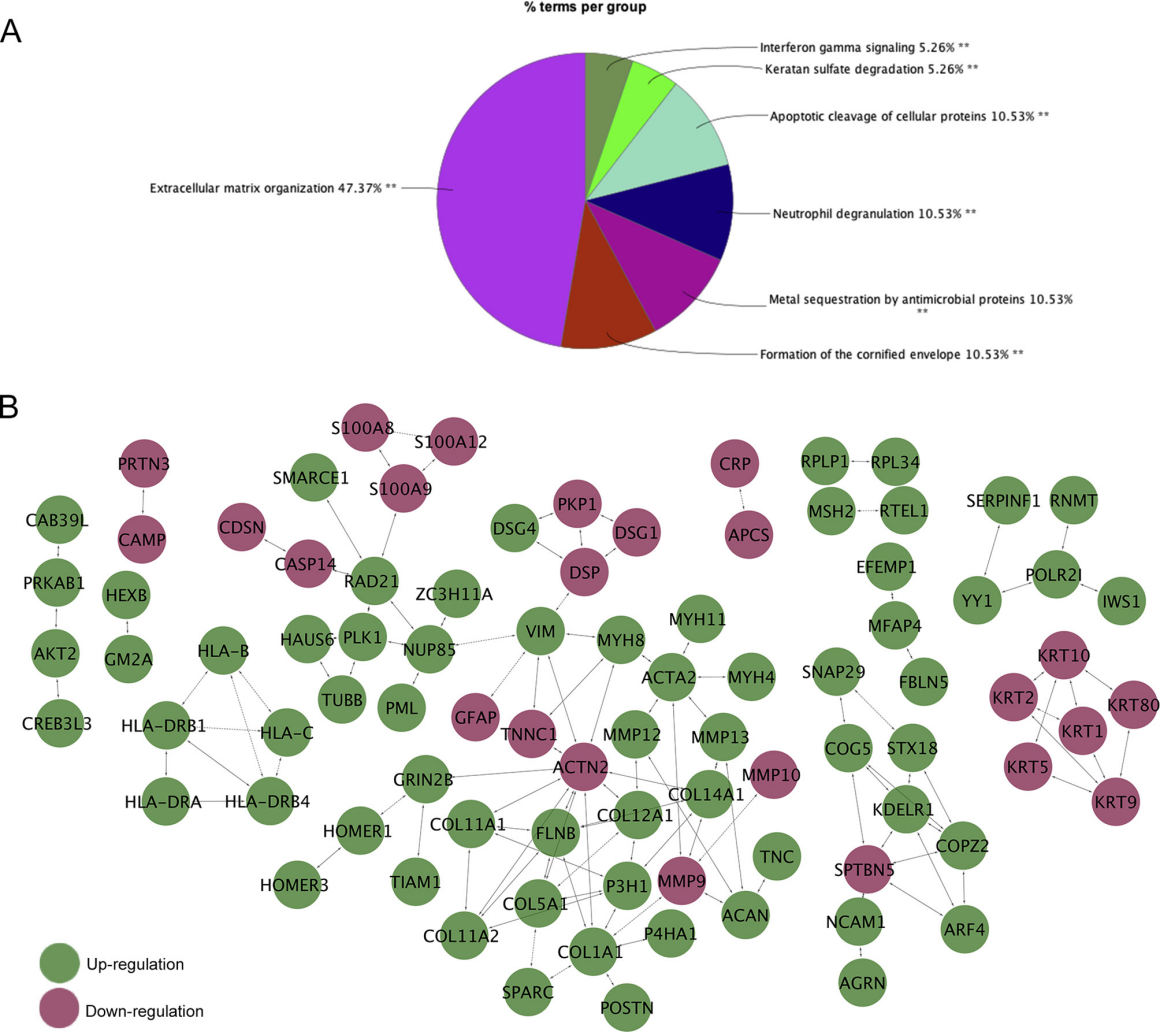
**Pathway analysis of the differentially-expressed proteins in tissue samples** The 281 differentially-expressed proteins between HO^−^ and HO^+^ tissue samples from the iTRAQ analysis were used as input for Cytoscape plugin, ReactomeFI, or ClueGO. **A.** Pie graph of the distribution for the non-redundant significant gene ontologies (two-sided hypergeometric test; ^**^P < 0.05). **B.** Clusters of protein–protein interactions for the differentially-expressed proteins (red and green indicates downregulation and upregulation in HO^+^ samples, respectively). Within each cluster multiple pathways are represented because of the involvement of proteins in many biological processes.

There were 82 and 281 differentially regulated proteins (down regulated ≤0.5; up-regulated ≥1.5) in serum and tissue, respectively. Among them, 7 proteins were differentially regulated in both serum and tissue samples. These include osteomodulin (OMD), collagen alpha-1(V) chain (COL5A1), macrophage-capping protein (CAPG), T-lymphoma invasion and metastasis-inducing protein 1 (TIAM1), C-reactive protein (CRP), serum amyloid A1 (SAA1), and SAA2. The 281 differentially expressed proteins from tissue samples are part of several Reactome pathways, which were significantly enriched (CLUEGO, *P* < 0.05). These include degradation of the extracellular matrix, striated muscle contraction, neutrophil degranulation, endosomal/vacuolar pathway, keratin sulfate degradation, apoptotic cleavage of cellular proteins, interferon gamma signaling, and latent infection of *Homo sapiens* with *Mycobacterium tuberculosis* (Figure 3A). Several direct protein–protein interactions (interactome) between the 281 differentially regulated biomarker candidates in tissue samples were revealed via ReactomeFI analysis (Figure 3B). Four pathways were enriched within the interactome with a false discovery rate (FDR) <0.001, including extracellular matrix organization, keratinization, neutrophil degranulation, and interferon gamma signaling. Interestingly, all 12 proteins identified in the keratinization pathway were down regulated in HO^+^ compared to HO^−^ samples.

### Validation of differentially-expressed proteins in individual samples

Individual HO^+^ and HO^−^ samples were tested using ELISA to validate potential markers that could be incorporated in a diagnostic panel for prospective studies. Utilizing the high throughput proteomic datasets and including proteins previously associated with HO in the literature, 26 proteins were analyzed in serum (Figure S1) of which 13 were measured in tissue as well (Figure S2). Five of the 26 proteins analyzed individually in serum samples were significantly different between HO^+^ and HO^−^ samples (Welch two sample *t*-test; *P* < 0.05), including insulin-like growth factor I (IGF1), osteopontin (OPN), myeloperoxidase (MPO), runt-related transcription factor 2 (RUNX2), and growth differentiation factor 2 or bone morphogenetic protein 9 (BMP-9) (Table 2). All proteins, except IGF-1, were down-regulated in HO^+^ compared to HO^−^ serum samples (Table 2). No significant differences were found for the 13 proteins examined in tissue homogenate, with interleukin 10 (IL-10) below detectable limits for all tissue samples. Of interest, BMP-2 showed a down-regulation trend in HO^+^ tissue samples relative to their HO^−^ samples (*P* = 0.060; Figure S2).

**Table 2:** **Proteins differentially expressed in serum samples between HO^+^ and HO^−^ subject cohorts**

**Protein name**	**UniProt ID**	**Gene**	***P* value**	**HO^+^**		**HO**^−^	
				**Mean (pg/ml)**	**Range (pg/ml)**	**Mean (pg/ml)**	**Range (pg/ml)**
Runt-related transcription factor 2	Q13950	RUNX2	0.002	1100.1	439.9–2160	2163.1	509.6–6038.5
Insulin-like growth factor I	P05019	IGF	0.008	92.5	45–139.8	56.4	3.6–115.6
Growth/differentiation factor 2	Q9UK05	BMP9	0.016	7.9	5.2–11.3	10.4	5.2–24.1
Myeloperoxidase	P05164	MPO	0.045	49.5	12.1–127.2	148.7	11.7–1081.4
Osteopontin	P10451	OPN	0.039	37.1	5.9–112.9	136.2	0.9–1215.2

### Integrative network analysis of differentially expressed proteins

**Figure 4:**
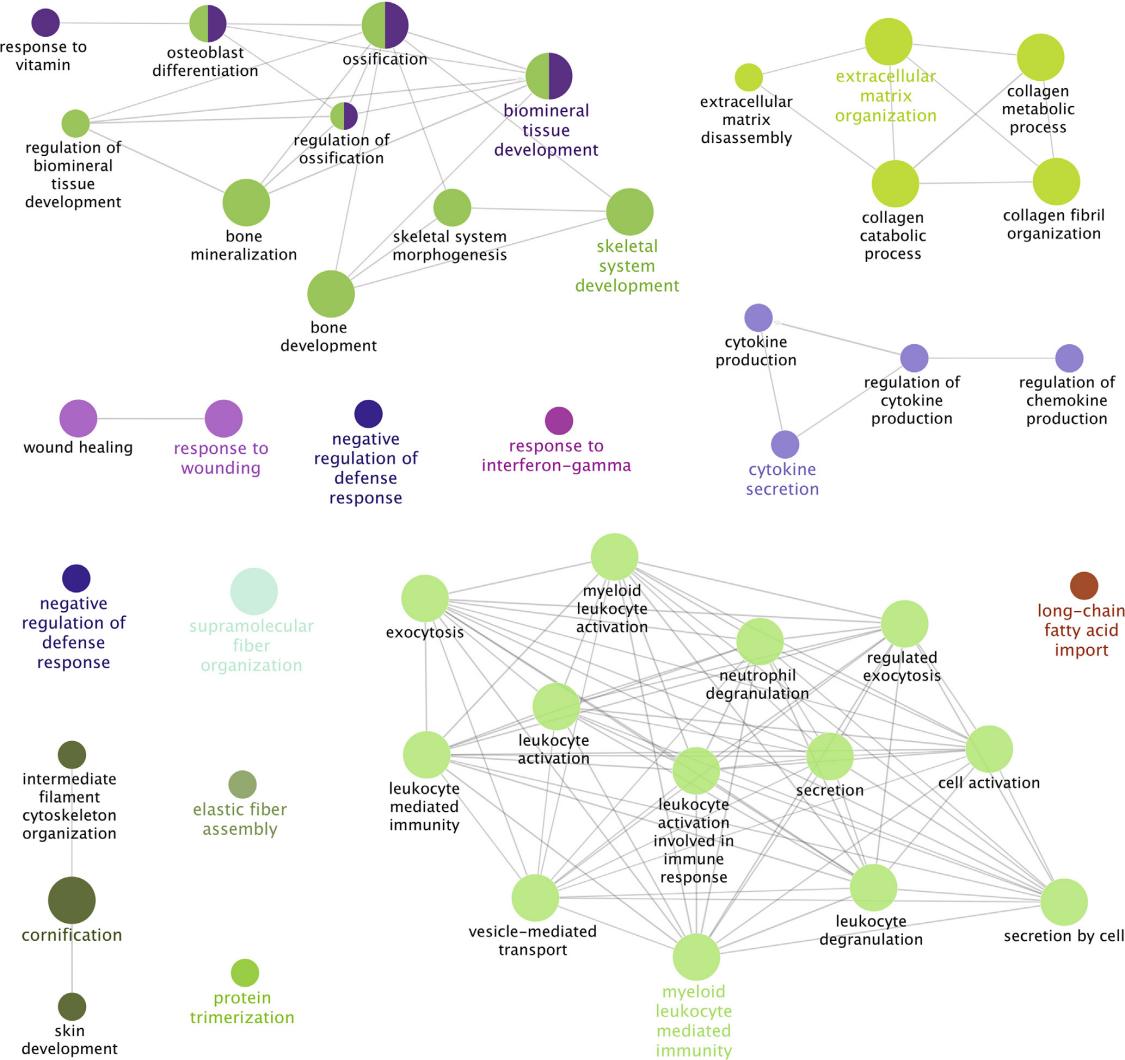
**Integrative network analysis of serum and tissue proteomic data for HO** Proteins that were differentially expressed either in serum (*n* = 90) or tissue (*n* = 281) samples between HO^+^ and HO^−^ cohorts, regardless of detection methodologies, were combined and analyzed collectively with ClueGO, and the significant non-redundant biological GO terms (*P* < 0.05) for the differentially-expressed proteins were displayed. The label and nodes of each cluster are colored by the most significant GO term in the cluster. Nodes showing 2 colors are included in multiple clusters. Node size reflects Kappa score. GO, gene ontology.

To integrate the data from the tissue and serum analyses, proteins that were differentially regulated between HO^+^ and HO^−^ cohorts, regardless of the detection methodology, were combined for further analyses of the biological pathways affected by HO. Proteins identified from serum samples include 5 proteins identified using ELISA, 82 proteins identified using iTRAQ and 3 proteins using SRM [7]. These 90 serum proteins were integrated with 281 tissue proteins identified using iTRAQ and analyzed using ClueGO [8], enabling visualization of the non-redundant biological terms for the differentially regulated proteins that can be grouped into networks (Figure 4). As a result, we identified 15 significantly different Reactome pathways (*P* < 0.05). These include biomineral tissue development, skeletal system development, response to fungus, antimicrobial humoral response, cytokine secretion, extracellular matrix organization, negative regulation of defense response, response to interferon-gamma, long-chain fatty acid import, elastic fiber assembly, supramolecular fiber organization, protein trimerization, cornification, response to wounding, and myeloid leukocyte mediated immunity. Among them, the four pathways biomineral tissue development, skeletal system development, extracellular matrix organization, and response to wounding represent biological processes relevant to HO disease, which manifests with abnormal bone growth.

## Discussion

Identification of biomarkers associated with HO is challenging because co-morbidities present with disease onset often confound analyses [9]. To better elucidate potential biomarker candidates specific to HO formation, a computational workflow was developed to integrate data from high-throughput and targeted proteomic assays of serum and tissue samples from HO^+^ and HO^−^ subjects. High-throughput, semi-quantitative, bottom-up, proteomic surveys do not require pre-determined targets and are therefore useful for discovery of potential biomarkers. Although quantitative antibody arrays quickly translate results into biological insight [10], the protein targets that can be included depend on antibody availability, sensitivity and specificity. Moreover, most site-specific post-translational modifications are not quantifiable using antibodies. To overcome these limitations, high-throughput MS approaches including iTRAQ and SRM were used in combination with antibody-based approaches. These techniques enabled a data driven experimental design and identified multiple differentially expressed proteins. These results suggest that HO is caused by the dysregulation of several cell signaling pathways and due to complex systemic and local interactions related to wound healing, as well as the recruitment of circulating progenitor cells [11].

Of the numerous proteomic analyses and findings for tissue and serum samples in the present study, five proteins were identified in individual serum samples as significantly different between HO^+^ and HO^−^ samples and show promise as potential clinical biomarkers. These include IGF1, OPN, MPO, RUNX2, and BMP-9. Expression of each of these proteins, except IGF-1, was down-regulated in HO^+^ compared to HO^−^ serum samples and all have a role in bone formation or inflammation. IGF-1 is involved in controlling bone mineralization and maturation (UniProtKB P05019) and is reported to enhance BMP-9 induced osteogenic differentiation in mesenchymal stem cells [12]. BMP-9 is a circulating inhibitor of angiogenesis that induces HO in damaged muscle [13] and can influence bone formation. MPO is a heme protein released by leukocytes that plays a significant role in inflammation and oxidative stress at the cellular level [14]. RUNX2 is a transcription factor involved in osteoblastic differentiation and skeletal morphogenesis (UniProtKB Q13950), which has been reported previously to be aberrantly expressed in HO [15]. OPN is a potent inhibitor of ectopic calcification and may influence inflammatory cell function at sites of ectopic calcification by dissolving minerals [11]. Osteoblasts produce high levels of OPN, which may connect osteoblasts with the apatite mineral of bone [16]. For each of these potential biomarkers, additional functional investigation is required to understand their specific role in the etiology of HO.

Both similar and contrasting results for protein abundance for some proteins identified in this workflow have been reported [12], [13], [14], [15], [16], [17], [18], [19], [20], [21], [22], [23]. Contrasting observations may be explained by the species studied (animal model or human), sample type (tissue, wound fluid, or serum), the time of sample collection post-injury, the type of target measured (protein or transcript), and/or the technique used to measure those targets. Furthermore, the use of tissue samples for biomarker discovery for HO is complicated by the difficulty identifying disease foci in sampled tissue, inability to sample repeatedly over time to monitor disease development and varied tissue types in samples. These factors are a source of variability for protein expression, as differences in protein abundance have been observed even within the same wound bed between the edge and the center of the wound [24].

In this study, both serum and tissue samples collected from the same subjects at the same time were analyzed for biomarkers of HO. The tissue and serum samples from subjects in this study represent a wide age range, and age is well known to negatively affect many biological processes including healing [25]. In addition, the sex ratio of HO^+^ and HO^−^ cohorts was not matched between or balanced within cohort. Sex-dependent differences in protein expression may be a confounding factor and source of heterogeneity. Although subjects were tracked after surgery for the development of HO, the tissue and serum samples were collected at the time of surgery and all HO^+^ samples were derived from subjects undergoing HO excision. Therefore, the samples analyzed in this study reflect serum and tissue status after HO onset rather than immediately post-injury as reported previously [13], [15], [17], [21], [22]. Typically, HO excision from tissues occurs 6 months or more after initial surgery to ensure maturation of the HO tissue, in the hopes of lowering the risk of recurrence or revision surgery. Understanding the dynamics of the transition from high expression of a protein after an injury to down-regulation over disease progression could provide insight into potential therapeutic targets.

Serum is a less-invasive sample compared to tissue and it can be collected at multiple and pre-determined time points. On the other hand, collection of tissue requires additional surgeries for a population already undergoing major surgeries including amputation and at risk for delayed healing due to extent of initial injuries. Additional surgeries may slow or prevent prosthetic use or return to functional ability. For these reasons, serum is more feasible than tissue for tracking HO onset and more uniform in composition for use in predictive diagnosis of the disease.

The advantages and disadvantages of shotgun proteomics compared to high-throughput antibody assays were evident in this study. Antibody assays are limited by availability of high quality antibodies (target specificity and limited cross-reactivity) for necessarily pre-determined protein targets of interest. The shotgun MS technique iTRAQ was able to overcome these disadvantages, but results are semi-quantitative.

To further transition iTRAQ findings into a clinical diagnostic panel, SRM-MS assays were used to quantitatively and robustly analyze serum peptides for 3 proteins, osteocalcin, osteomodulin, and collagen alpha-1(v) chain isoform 2 [7]; and quantitative antibody assays were used to measure 5 proteins: IGF1, OPN, MPO, RUNX2, and BMP-9, which are all potential clinical biomarkers for HO. Collectively, these 8 proteins are candidates for a serum diagnostic panel that, once validated, may detect onset of HO.

In conclusion, our results support that a multi-protein longitudinal assay is required for an effective biomarker panel for HO. Linking these biomarkers to potential new therapies is essential for improving patient outcomes. Our data support that multiple cell signaling pathways in both serum [7] and tissue, including extracellular matrix organization and keratinization, are dysregulated in subjects that develop HO and are potential cellular processes to target. Ultimately, as precision medicine efforts continue to drive the use of advanced technologies, including LC-MS/MS and next generation sequencing, a multi-protein panel approach, coupled to advanced analytics, is a clinically relevant and viable diagnostic platform for detection and monitoring of HO.

## Materials and methods

### Subject enrollment

Subjects under treatment for high-risk fractures, acetabular fractures, burns with orthopedic injury, traumatic brain injury with extremity trauma, amputation, excision of ectopic bone, or major arthroplasty were enrolled into this study. Subjects aged ≤18 years or subjects being treated for cancers or metastatic disease involving bone were excluded. All subjects were enrolled prior to surgery. Surgical procedures included total hip arthroplasty, open reduction and internal fixation, hip revision, incision and drainage below knee amputation, intramedullary nailing, and HO excision. The HO^−^ tissue samples were acquired mainly through total hip arthroplasty, whereas the HO^+^ samples were acquired via hip revision or HO excision. Disease state, HO^+^ or HO^−^, was determined by evaluation of radiographs that were collected at time of surgery, and follow-up visits at 6 weeks, 12 weeks, 6 months, and 12 months. Written informed consent was obtained from all subjects.

### Sample collection and processing

Blood (5cc) and tissue samples were collected, at a single time point, during the surgical procedure at the same time. Serum was processed and snap frozen in liquid nitrogen and stored at −80 °C prior to use as previously reported [7]. Tissue removed at surgery was placed in a sterile 50-ml conical tube and stored at −80 °C until protein extraction.

Frozen tissue was thawed on ice and 1 g (wet weight) was collected into a sterile petri dish and minced with scalpel blade. Tissue was homogenized in 15 ml ice-chilled lysis buffer (http://www.kinexus.ca/ourServices/microarrays/antibody_microarrays/details/index.html; Kinexus, Vancouver, BC, Canada) containing EDTA-free 1 mM dithiothreitol (DTT) and 1× protease inhibitor inhibitors (HALT™, ThermoFisher Scientific, Waltham, MA) in a T10 115 V Disperser (coupled with an S10N-10G stainless steel dispersing element) with 3–5 repeated 15-sec pulses and sonicated (Ultrasonic Processor, Model GEX130, IKA-Works, Inc., Wilmington, NC) (130 W) with 4 repeated 10-sec pulses at 50% amplitude. Samples were centrifuged (Hettich MIKRO 220R, Andreas Hettich GmbH & Co.KG (Hettich), Tuttlingen, Germany) first at 6000 × rcf g for 10 min × 2 at 4 °C to pellet tissue debris and then at 14,000 × rcf g for 30 min at 4 °C. Protein concentration of the resulting supernatant was determined in duplicate using the Bradford method (Catalog No. 500-0006, BioRad, Hercules, CA) in microplate format with bovine IgG (Catalog No. 500-0005, BioRad) as the standard. Supernatants were aliquoted into 1.5-ml Protein LoBind Eppendorf tubes (Eppendorf 02243108, Hamburg, Germany) and stored at −80 °C.

### Antibody microarray

HO^−^ and HO^+^ samples, from 4 subjects, were pooled separately and screened using an 877 target antibody microarray according to the manufacturer’s specifications (catalog No. KAM-850, Kinexus). Candidate biomarkers were selected considering: (1) the degree of change observed between HO^−^ and HO^+^ pools with ±50% clash free crossovers (CFC); (2) the intensity of the globally normalized signal intensity score >800; (3) the sum of the % error range in the duplicate measurements for each HO^−^ and HO^+^ antibody pair and their comparison to the % CFC value (set as sum <80% of CFC value); and (4) ranked Z-ratio scores.

### Mass spectrometry

Equal amounts of protein from each subject were pooled according to the disease status. Thirty-one HO^−^ and 10 HO^+^ serum samples, as well as 33 HO^−^ and 9 HO^+^ tissue samples were included for mass spectrometry analysis as previously reported [7]. The average labeling efficiency of all iTRAQ quantitative channels (MyOmicsDx, Towson, MD) was 99.2% through Proteome Discoverer (version 2.2) with a repetitive random sampling of 1000 peptides from the entire quantified peptidome.

### Quantitative antibody assay

Protein abundance in serum and tissue homogenates was measured in duplicate by commercial assay services (Assaygate, Ijamsville, MD) using bead-based multiplex suspension arrays with the Bio-Plex 200 Bead Reader System or conventional solid phase sandwich enzyme linked immunoassay (ELISA). Thirteen targets were measured in both serum and tissue homogenates, procalcitonin, matrix metalloproteinase-9 (MMP-9), stromal cell-derived factor 1 (SDF-1a), transforming growth factor beta-2 (TGFβ2), bone morphogenetic protein 2 (BMP-2), BMP-9, BMP-4, interleukin-6 (IL-6), tumor necrosis factor alpha (TNF-a), macrophage inflammatory protein 1-alpha (MIP-1a), monocyte chemoattractant protein-1 (MCP-1), IL-10, and C-X-C motif chemokine 10 (CXCL10). An additional 13 targets were measured in serum samples only, cartilage oligomeric matrix protein (COMP), IGF1, thrombospondin-1 (THBS1), pro-epidermal growth factor (EGF), OPN, MPO, secreted protein acidic and rich in cysteine (SPARC), TGFβ1, RUNX2, talin-1 (TLN1), plasminogen, fibroblast growth factor receptor 1 (FGFR1), and gremlin-1. Differences in protein concentration between HO^+^ and HO^−^ samples were tested using Welch two-sample *t*-test with significance for *P* < 0.05.

### Pathway and gene ontology analysis

Bioinformatics analyses of iTRAQ data were performed using Cytoscape v3.3.0 [26] and the ReactomeFI plugin (database 2016) [27]. Gene ontology enrichment analysis was performed using the Cytoscape applications BiNGO [28], REVIGO [29], and ClueGO [8]. BiNGO parameters were as following: over presentation of gene ontologies after FDR correction, hypergeometric test, Benjamini–Hochberg FDR correction, *P* < 0.05, and GO: biological processes. BiNGO results were up-loaded into REVIGO and analyzed using default settings. ClueGO parameters were as following: ClueGO functions, Reactome pathways, *P* < 0.05, enrichment/depletion (two-sided hypergeometric test), and prefuse force directed layout.

## Authors’ contributions

LEE conceived the study and participated in its design and coordination. JTW participated in the study design and bioinformatics analyses. ELC carried out the bioinformatics analyses. PMO was involved in the sample collection and TTW acquired clinical data. LEE, ELC, and JTW drafted the manuscript. All authors revised the manuscript. All authors read and approved the final manuscript.

## Competing interests

The authors declare that they have no competing interests.
