# Supplementary figures and images for: A Systems Biology Approach for Studying Heterotopic Ossification: Proteomic Analysis of Clinical Serum and Tissue Samples
Source: Genomics Proteomics Bioinformatics. 2018 Jul 24;16(3):212–20. doi: 10.1016/j.gpb.2018.04.006 (PMC6076384; doi:10.1016/j.gpb.2018.04.006)

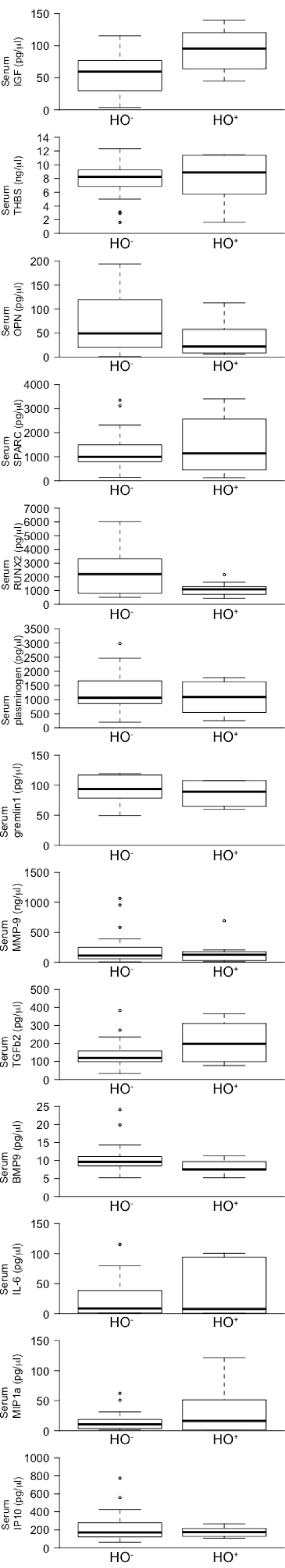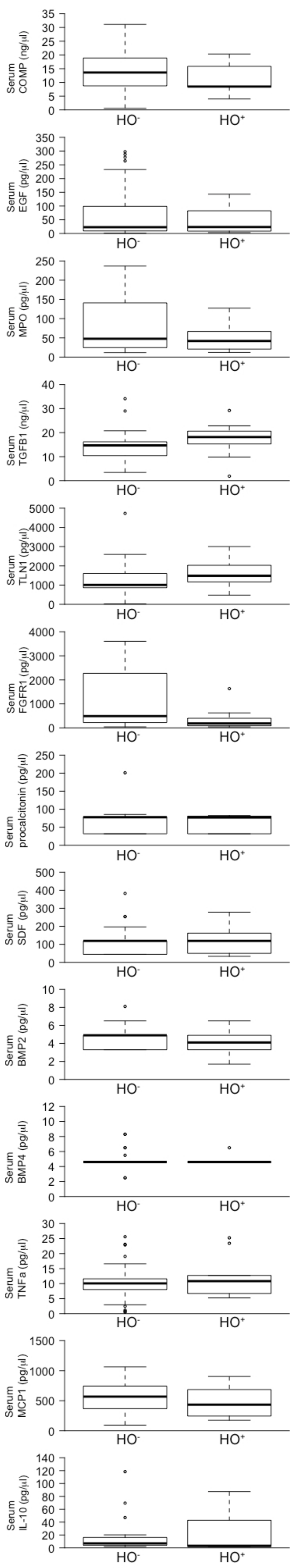

Supplement: Supplementary Figure S1 [file mmc1.pdf]

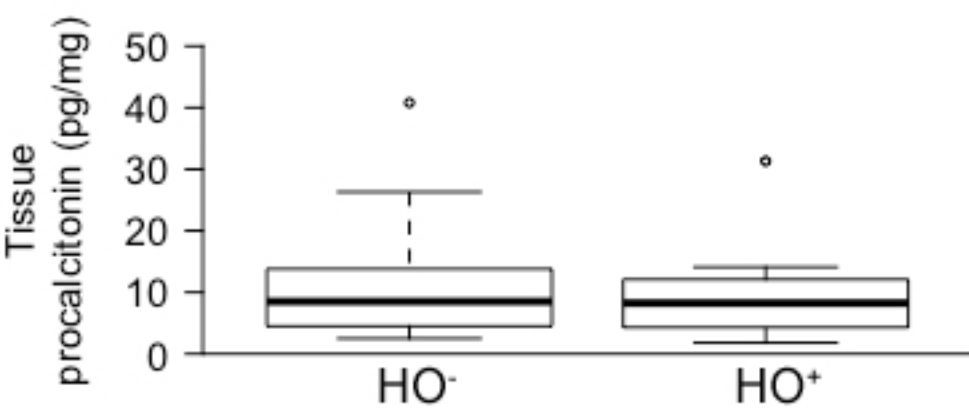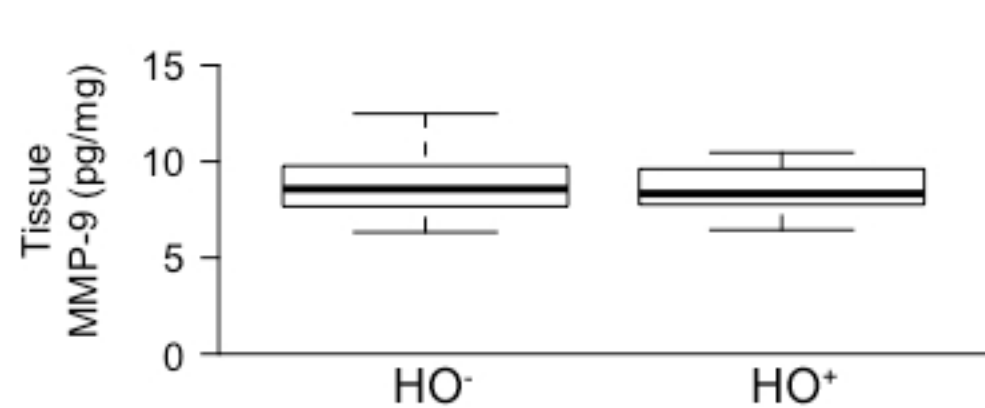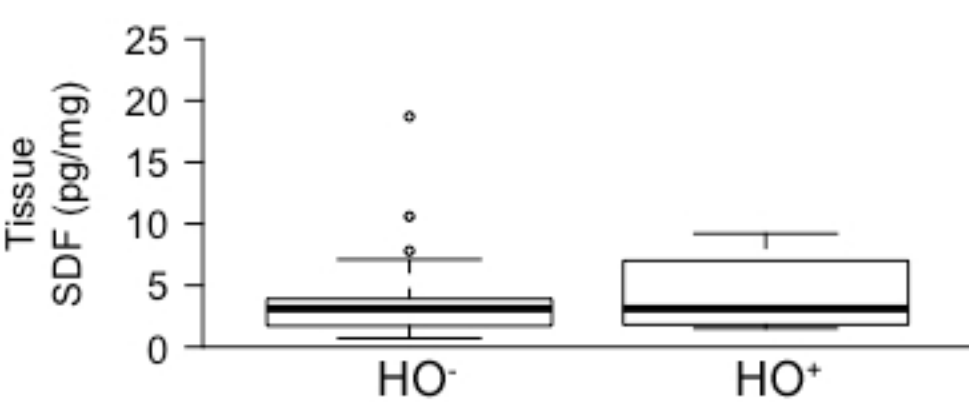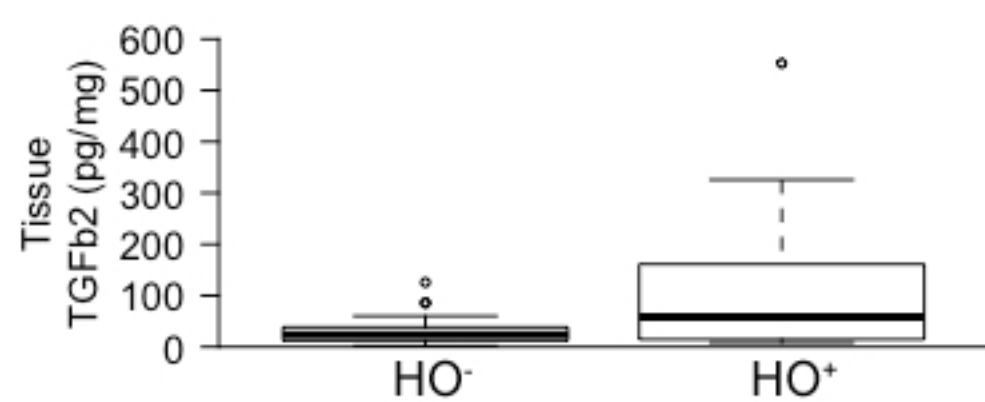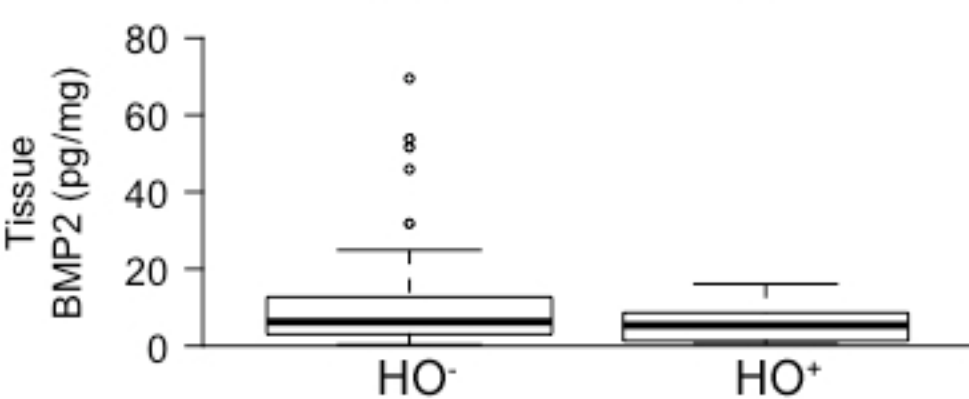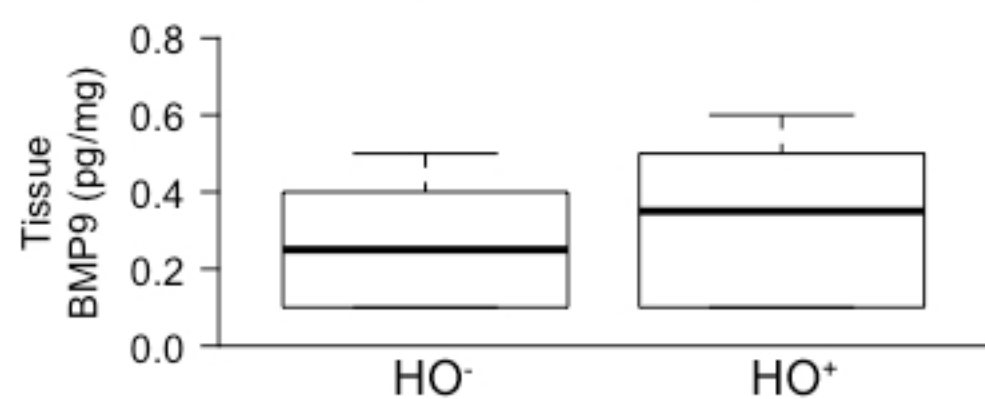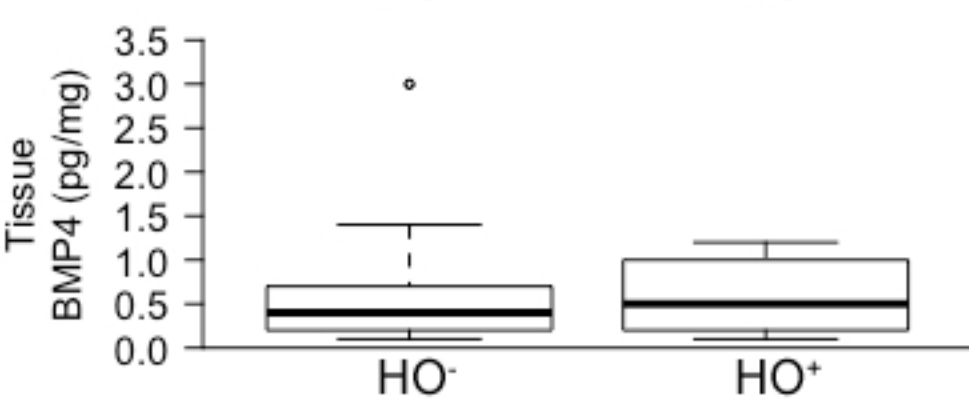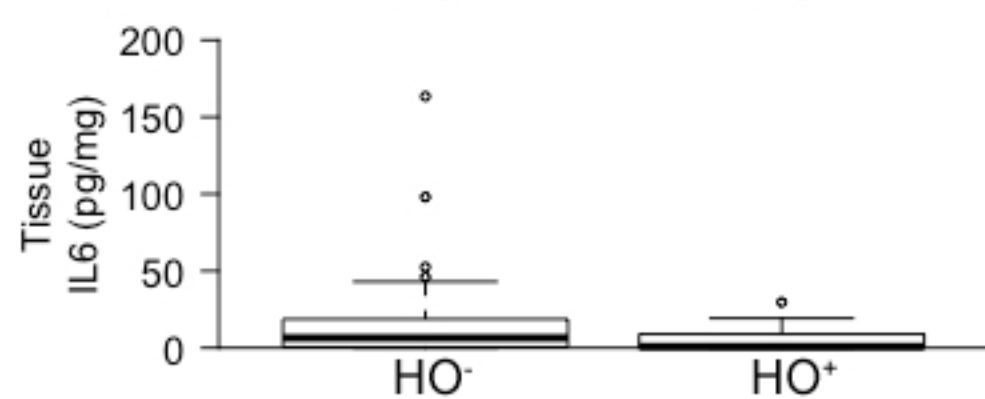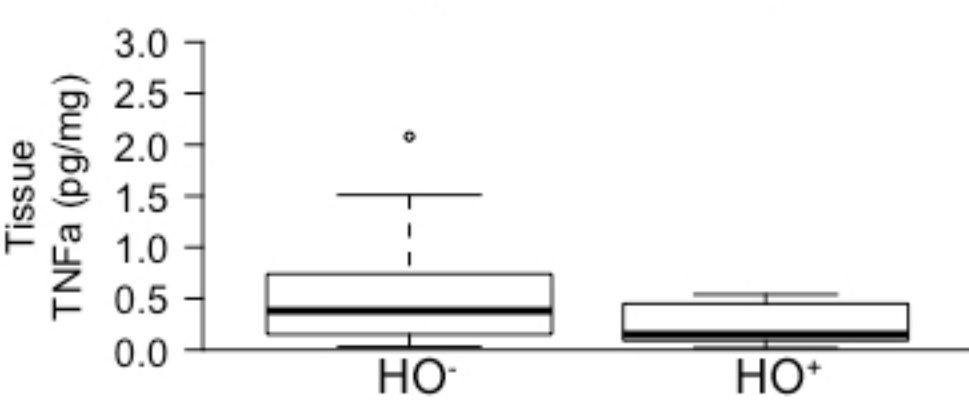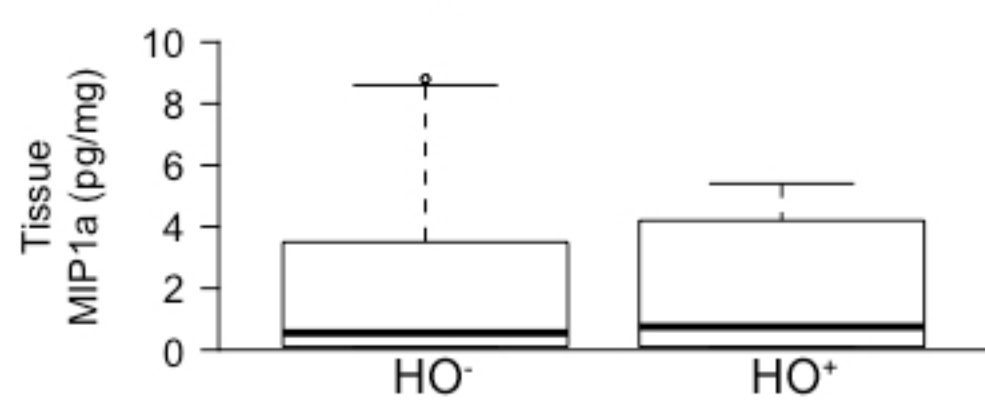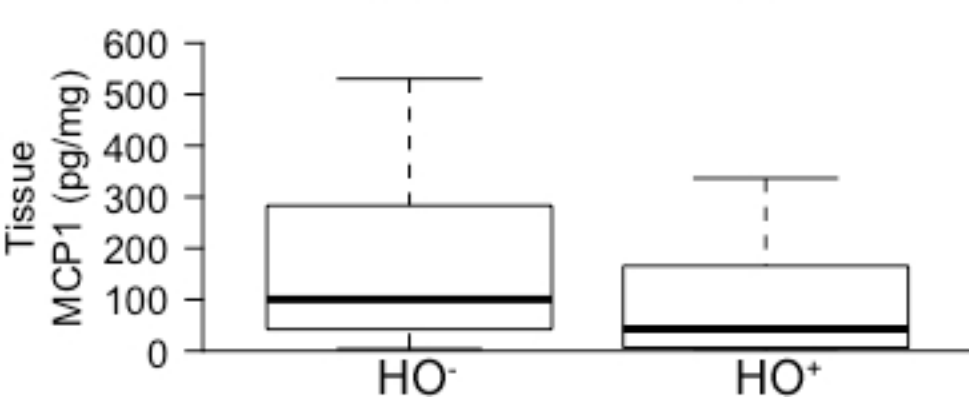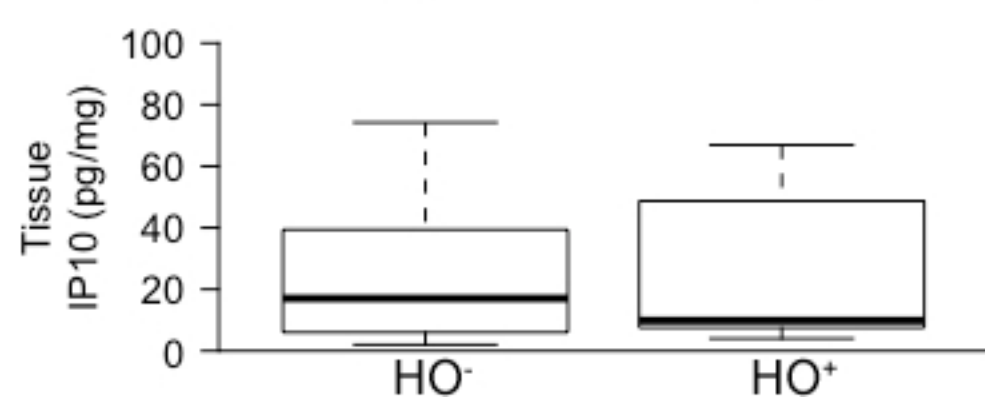

Supplement: Supplementary Figure S2 [file mmc2.pdf]
